# Learning by Insight-Like Sudden Comprehension as a Potential Strategy to Improve Memory Encoding in Older Adults

**DOI:** 10.3389/fnagi.2021.661346

**Published:** 2021-06-14

**Authors:** Jasmin M. Kizilirmak, Larissa Fischer, Justus Krause, Joram Soch, Anni Richter, Björn H. Schott

**Affiliations:** ^1^German Center for Neurodegenerative Diseases, Göttingen, Germany; ^2^Institute of Psychology, University of Hildesheim, Hildesheim, Germany; ^3^Leibniz Institute for Neurobiology, Magdeburg, Germany; ^4^Bernstein Center for Computational Neuroscience, Berlin, Germany; ^5^Department of Psychiatry and Psychotherapy, University Medical Center Göttingen, Göttingen, Germany

**Keywords:** long-term memory, aging, problem solving, insight, learning, memory formation

## Abstract

Several cognitive functions show a decline with advanced age, most prominently episodic memory. Problem-solving by insight represents a special associative form of problem-solving that has previously been shown to facilitate long-term memory formation. Recent neuroimaging evidence suggests that the encoding network involved in insight-based memory formation is largely hippocampus-independent. This may represent a potential advantage in older adults, as the hippocampus is one of the earliest brain structures to show age-related volume loss and functional impairment. Here, we investigated the potential beneficial effects of learning by insight in healthy older (60–79 years) compared to young adults (19–28 years). To this end, we compared later memory performance for verbal riddles encoded incidentally via induced insight-like sudden comprehension in both age groups. We employed a variant of the Compound Remote Associate Task (CRAT) for incidental encoding, during which participants were instructed to judge the solvability of items. In a 24-h delayed surprise memory test, participants attempted to solve previously encountered items and additionally performed a recognition memory test. During this test, older adults correctly solved an equal proportion of new CRA items compared to young adults and both age groups reported a similar frequency of Aha! experiences. While overall memory performance was better in young participants (higher proportion of correctly solved and correctly recognized old CRA items), older participants exhibited a stronger beneficial effect of insight-like sudden comprehension on later recognition memory for CRA items. Our results suggest that learning via insight might constitute a promising approach to improve memory function in old age.

## Introduction

Human cognitive functioning is subject to considerable alterations during aging, most prominently with regard to memory function. However, not all types of memory are affected to the same degree by age. Episodic memory, particularly explicit episodic memory encoding, typically shows the most pronounced decline in older adults, particularly from 67 years onward (Rönnlund et al., 2005; Nyberg et al., 2012). Although some cross-sectional studies suggest a linear decline, longitudinal studies, which evade cohort-effects like different generation-dependent educational backgrounds, show that episodic memory remains stable for a long time, before it begins to decline around the mid-60ies (Zelinski and Burnight, 1997). The specific age-related cognitive changes are strongly related to changes in the brain. The medial temporal lobe (MTL), encompassing the hippocampus, perirhinal and entorhinal cortex, and the parahippocampal cortex, is known to be crucial for episodic memory encoding and retrieval (e.g., Squire, 1992; Squire et al., 2004; Gold et al., 2006), until the information has become semanticized (Bonnici et al., 2012; Sommer, 2017). The high dependence of episodic memory on the integrity of the hippocampus poses a problem at a higher age (Nyberg et al., 2012), as the MTL is one of the first regions to show age-related volume loss and functional impairment (e.g., Fjell et al., 2009; Craik and Rose, 2012). Another reason for age-related differences especially with regard to episodic encoding has been proposed in a review by Craik and Rose (2012). Younger and older adults appear to differ considerably in their use of attentional resources as well as their active semantic elaboration of novel information, which has been associated with age-related structural and functional alterations in lateral fronto-temporal regions like the inferior prefrontal cortex^1^ (attentional selection) and anterior temporal lobe (semantic integration). This has profound consequences for memory performance as it affects the level-of-processing of novel information and thus efficiency of encoding (Craik and Lockhart, 1972). However, older adults can be steered to employ encoding strategies that use existing neural resources more efficiently. *Incidental encoding* (unintentional, automatic learning) and *relying on existing semantic knowledge* are two ways to facilitate learning at an advanced age (Troyer et al., 2006; Wagnon et al., 2019). For example, when older adults are encouraged to use deeper levels of encoding by actively making semantic decisions about novel information, later memory performance can be improved (Grady and Craik, 2000).

In a thus far largely separate line of research, the phenomenon of insight from the problem-solving domain has attracted the attention of memory researchers, because it appears to unite incidental encoding, prior-knowledge-related encoding and a deep level of processing: learning by insight. Insight can be defined as sudden comprehension that overcomes a previous state of incomprehension (Auble et al., 1979). It can essentially be considered a discontinuous problem-solving process (Zander et al., 2016), during which initial attempts at problem solving are unsuccessful, followed by a sudden understanding of a novel relationship between the pieces of the puzzle and prior knowledge that make up the solution (Ohlsson, 1984a,b; Ohlsson, 1992). It has further been shown that the feeling of Aha! can both be evoked by solutions found by the participant as well as by those presented by the experimenter, so-called *induced insight* (Kizilirmak et al., 2016a,c).

While Gestalt psychologists like Wolfgang Köhler have suggested over a century ago that learning may be facilitated when novel relationships are comprehended suddenly, by insight (Köhler, 1917), empirical research specifically directed at the potential beneficial effect of insight on memory encoding only started less than two decades ago (Ash et al., 2009; Ludmer et al., 2011; Danek et al., 2013; Kizilirmak et al., 2016a). It has been shown that later memory performance can be increased for incidental encoding with insight compared to encoding without insight (Danek et al., 2013; Kizilirmak et al., 2016a). The neurocognitive basis of this memory advantage is a topic of ongoing investigation (Danek and Wiley, 2020). Current research suggests that it is based on a combination of *cognitive* and *affective* aspects. A *cognitive* aspect is the *generation effect* (Slamecka and Graf, 1978), which reflects a combination of a deep *level-of-processing* (Craik and Lockhart, 1972) and activation of prior knowledge which a novel piece of information (i.e., the solution) can be linked to van Kesteren et al. (2014). *Affective* components are, for example, intrinsic reward or the feeling of certainty for the solution being correct that are part of the subjective Aha! experience (Danek et al., 2014; Kizilirmak et al., 2016a,c; Danek and Wiley, 2020).

In other words, learning by insight appears to represent a mechanism that simultaneously promotes multiple neurocognitive processes that can enhance memory encoding in older adults. Recent neuroscientific studies in animals and humans have shown that the hippocampus plays a much smaller role in learning novel information when this information can be readily integrated into a framework of pre-existing knowledge (so-called schemas) (Tse et al., 2011; van Kesteren et al., 2012; Brod et al., 2013). Learning via insight appears to represent such a special way of incidental, associative learning that is largely hippocampus-independent (Kizilirmak et al., 2016b, 2019). The question arises whether learning by insight represents a possibility to facilitate learning in older adults by drawing on these extant resources.

**FIGURE 1:**
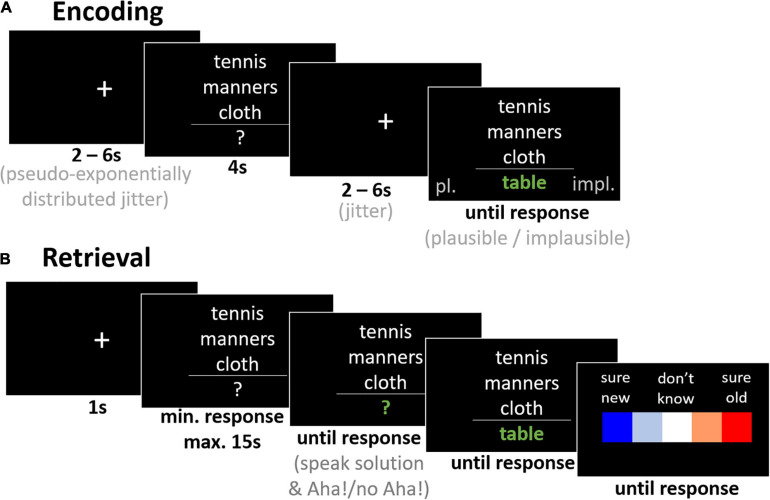
Exemplary trials for the incidental encoding task **(A)** and the memory test **(B)**.

In the present study, we tested this hypothesis by comparing healthy young (19–28 years) and older adults (60–79 years) with regard to later memory performance after incidental learning via insight-like sudden comprehension. To this end, we employed a variant of the Compound Remote Associate Task (CRAT), which has previously been adapted for the study of insight-related memory by our group (Kizilirmak et al., 2016c). Participants were presented with verbal riddles during encoding (see Figure 1) and were to judge the plausibility of the presented solution. The items were either solvable CRA items or unsolvable control items. The presentation of the solution to solvable items was intended to induce insight-like sudden comprehension, while the presentation of pseudo-solutions to unsolvable items should evoke continued incomprehension for comparison. In a 24 h delayed memory test, participants attempted to solve previously encountered (*old^2^*) and *new* CRA and control items, followed by an old/new recognition memory decision. We assessed memory performance by means of solution rates of *old* and *new* items as an indirect measure of memory, and by means of an old/new recognition decision to assess the discrimination. The advantage of indirect memory tests is that even when participants do not explicitly remember having processed an item previously, higher solution rates can be expected for *old* items even for implicit learning (Richardson-Klavehn, 2010). Thus, semantic encoding success can be tested independently of explicit recognition, which is especially advantageous for older participants, as explicit recollection shows earlier age-related decline (Tromp et al., 2015).

Our main hypothesis was that (1) learning by induced insight-like sudden comprehension would facilitate learning in older adults to a higher degree than in the young. This should be reflected by a larger difference in the discrimination rate for CRA compared to control items in older adults, and larger solution rates for *old* versus *new* CRA items as compared to young adults. Additionally, due to their larger vocabulary, (2) older adults should be more accurate in deciding on the plausibility of a presented solution during encoding. Similarly, following a study showing a broader semantic knowledge of older compared to young participants (Privodnova and Volf, 2016), we further hypothesized that (3) older adults may have an advantage in solving *new* items during the memory test, reflected by higher solution rates for *new* CRA items. On the other hand, the young have higher cognitive flexibility and can access their knowledge faster in addition to faster average reaction times (e.g., Kray and Lindenberger, 2000). This may lead to a balance, resulting in similar solution rates.

As this is, to our knowledge, the first study to assess learning by induced insight in participants of advanced age, we also looked into a set of exploratory questions: (4) Are there age-related differences in the frequency of Aha! experiences reported for true and false insights, i.e., correct and incorrect solutions? Generally, Aha! experiences are reported more often for correctly solved items (true insights) as compared to incorrect solutions (false insights) (Danek and Wiley, 2017; Danek and Salvi, 2018). (5) Are there age-related differences in which phenomenological aspects of the Aha! experience receive a higher weight for deciding on whether an Aha! moment occurred?

## Materials and Methods

### Participants

We investigated 61 healthy volunteers^3^ of two age groups: 30 young participants aged between 19 and 28 years, and 31 older adults aged 60–79 years. The older group had a median age of 67 years (*M* = 66.81, *SD* = 4.43), the younger group had a median age of 23 years (*M* = 22.77, *SD* = 2.76). All participants were native speakers of German, as the verbal riddles were in German. Detailed demographic data are provided in Table 1. All had sufficient visual acuity, either naturally or by using a visual aid, to read the words on the screen without difficulty, and reported no history of neurological diseases or mental disorders. We conducted a dementia screening (Mini-Mental Status Examination, MMSE; Folstein et al., 1975; Beyermann et al., 2013), a verbal intelligence screening (MWT-B; Lehrl, 1999), and a general health questionnaire in all participants. All participants could be included in the final sample for data analysis. None of the participants had an MMST score below 24, the widely accepted cut-off value for dementia (Creavin et al., 2016). Participants received financial compensation or course credits (for students) after completion of the experiment. Thirty-one (16 older, 15 young) of the participants were tested at the University of Hildesheim and 30 (15 older, 15 young) were tested at the Leibniz Institute for Neurobiology (LIN) Magdeburg.

**TABLE 1:** Demographic data of all participants included in the study and analyses.

Group	*N*	Age (years)	Gender^a^	Handedness^a^	MMSE^b^	MWT-B^b^	Highest education^a^
Older	31	66.81 (4.43)	f: 19 m: 11 d: 1	Right: 30 Left^c^: 1 (re-educated)	28.52 (1.18)	31.42 (2.45)	Junior High: 10 High School: 2 Master: 19
Young	30	22.77 (2.76)	f: 22 m: 8	Right: 25 Left: 5	29.1 (0.96)	25.03 (2.24)	High School: 23 Bachelor: 6 Master: 1

*This table does not report participants by site, because no site effects were observed, except for reaction time (see Section “Congruency of Plausibility Decision and Response Times”). ^*a*^According to self-report. ^*b*^Mean score with standard deviation in parentheses. ^c^Including left-handed participants is unproblematic, because we assess within-subjects manipulations, i.e., each participant is compared with themselves, and no neuroimaging data were acquired.*

Participants were recruited via university mailing lists and social media, via a newspaper article in Hildesheim, and via an existing participant pool of an ongoing study on age-related cognitive changes at the LIN Magdeburg (Assmann et al., 2020; Soch et al., 2021a,b). In Hildesheim, young participants were mainly recruited via digital media and older participants via newspaper call. In Magdeburg, both age groups were equally represented in the existing participant pool at the LIN. The data were collected between November 2019 and January 2020.

The study was approved by the Ethics Committees of the Department of Education at the University of Hildesheim and of the Otto von Guericke University of Magdeburg, Faculty of Medicine. Before the start of the experiment, the participants were informed about the procedure and their rights regarding data protection in accordance with European, federal, and state data protection regulations and gave their written consent to participate in the study in accordance with the Declaration of Helsinki (World Medical Association, 2013).

### Stimulus Material

We used a total set of 180 German CRA items, created and employed previously by our group (Kizilirmak et al., 2016a,b, 2018). Each item consists of three nouns or color words (triad) and a fourth solution word. The *solution* could be used to create compound words with each of the other three (e.g., tree *stem*, brain *stem*, *stem* cabbage). About half of all items were homogeneous (solution word can be used as prefix or suffix for all triad words) and half were heterogeneous (solution word can be used as prefix/suffix for 1–2 triad words).

For each participant, a subset of 140 items, consisting of 60 *old* CRA items, 30 *new* CRA items, 30 *old* control items, 15 new control items, and 5 practice CRA items, was chosen in the following manner: (1) Six sublists of 30 solvable CRA items each (lists: A, B, C, D, E, F) were created that were matched with regard to solution rate, plausibility rating, and probability of an Aha! experience, which was derived from an unpublished pilot with 20 subjects. (2) The six sublists were used to create six lists of 30 unsolvable control items each (A_shuffled, B_shuffled, etc.). To this end, all triad words and all solution words per list were shuffled separately and newly combined, using MATLAB’s randperm() function, followed by manual inspection to ensure that thus created control items were indeed implausible. Note that the words composing a triad did not stay together, but were themselves mixed with words from the other triads. (3) According to a *reduced Latin square* (Kempthorne, 1952), for each subject we assigned two of the solvable CRA item lists that would be shown during encoding and testing (*old* CRA), one sublist for unsolvable control items for encoding and testing (*old* control), one sublist for *new* CRA items only to be used during memory test, the first half of one sublist for *new* control items, and of the remaining list, we took the first five items to be used as solvable practice items. For example, for one participant the list was A, B, C_shuffled, D, E_shuffled and items 1 to 5 of list F for practice. For the next participant, it was B, C, D_shuffled, E, F_shuffled and items 1–5 of list A for practice, and so on.

### Task and Procedure

Exemplary trials of the encoding and memory test sessions are depicted in Figure 1. The experiment consisted of two experimental sessions. On day 1, participants were informed about the procedure and gave written informed consent, before the MMSE, the MWT-B, and the health questionnaire were administered. Afterward, participants performed the encoding session at a desktop PC after receiving a written task instruction, which they were asked to summarize orally for the experimenter to assess whether the task was understood correctly. On day 2, participants performed the memory test at the PC after having again received written instructions. These task instructions also included a definition of the Aha! experience as described in Kizilirmak et al. (2019), covering the criteria of suddenness, feelings of ease, confidence in the correctness of the solution, and positive affect, as described by Topolinski and Reber (2010). During incidental encoding on day 1, the timeout was too brief for participants to find a solution (4 s), which enabled us to induce sudden comprehension by presenting the solution. On day 2, participants had enough time to actually solve some items themselves (15 s), thereby allowing to assess solution rates of *old* versus *new* items as an indirect measure of memory. Moreover, when an item was solved by the participant, we asked them whether they had had an Aha! experience as described in the task instructions they had received beforehand. After the memory test, participants filled out a post-experimental questionnaire to assess potential confounding variables, such as use of strategies, participants’ ideas about our foci of investigation, whether they solve riddles in their leisure time, and whether they had a main criterion when providing the Aha! experience decision after they had solved an item.

The encoding session on day 1 started with five practice trials during which the experimenter made sure that participants had correctly comprehended the task. Then, 60 solvable CRA items and 30 unsolvable control items were presented. Each trial started with a fixation cross in the center of the screen (white on black) for 2 to 6 s (chosen with a pseudo-exponential distribution). This interval was used as this paradigm is intended to be used with functional neuroimaging in a future study, and we wanted to know exactly what kind of behavioral performance we could expect. Following fixation, a verbal riddle was presented for 4 s (see Figure 1A). Three words were presented stacked and centered. A horizontal line separated the triad from a question mark that was the placeholder for the solution word. Item order was shuffled individually for each participant. Participants were encouraged to start searching for the solution themselves, to help them make a plausibility judgment on the solution as soon as it was presented. In this regard, the written instructions they had received earlier read (translated from German): “The puzzles are displayed only briefly. There are solvable and unsolvable puzzles. We want to know, if you can distinguish between them. You can do this better and faster, if you attempted to solve the puzzle first. However, due to the very short presentation time, you will probably only be able to solve very few puzzles. When you have found a solution, please press the space bar immediately. Note that despite pressing the key, the puzzle will continue to be displayed on the screen until the time runs out.” This plausibility decision was followed by another inter-stimulus interval of 2–6 s, during which a fixation cross was presented. The solution was then presented together with the triad until a response was made via button press. The task was to judge the plausibility as a binary decision (plausible/implausible). Button assignment to decision (left and right arrow keys, pressed with index and ring finger of right hand, to plausible/implausible) was counterbalanced across participants to avoid confounding of responses to dominant fingers (typically the index finger).

On day 2 (24 h later, ±1 h), memory was assessed as follows: Participants attempted to solve *old* (had been presented on day 1) and *new* items. A total of 135 items (60 solvable *old* CRA items, 30 solvable *new* CRA items, 30 unsolvable *old* control items, and 15 unsolvable *new* items) were presented. Each trial started with a white fixation cross on black background, presented for 1 s (see Figure 1B). The triad, i.e., three words presented stacked in the center of the screen, with a question mark below a line directly underneath, was presented directly after, either until button press (*space bar* to indicate that the riddle had been solved) or until the timeout of 15 s was reached. As soon as the button was pressed, this display was immediately replaced by an identical one in which the question mark changed color to green to indicate that it was okay to say aloud the solution and whether participants had experienced an Aha! moment. Both oral responses were written down by the experimenter. After pressing *space* again, the trial continued with a display of the triad plus the correct solution. This display remained until button press to ensure that participants had read and understood the solution even if they had not solved it themselves (especially for control items which could not be solved). The last display contained a 5-point scale from *sure new* (–2), over *probably new* (–1), *don’t know* (0), to *probably old* (1), and *sure old* (2), which remained on screen until participants had chosen one of the values via arrow keys and confirmed by pressing *space*.

### Experimental Design

We used a 2 × 2 × 2 mixed factorial design with *Age* as between-subjects factor (young vs. older participants) and the within-subjects factors *Condition* (solvable vs. unsolvable) and *Stimulus* (*old* vs. new*).* The experiment consisted of two sessions: incidental encoding on day 1 and memory testing on day 2, 24 ± 1 h later. During encoding, participants judged the plausibility of items presented with correct (solvable CRA items) or pseudo-solutions (unsolvable control items), presented in random order. By presenting correct solutions for the CRA items, we intended to induce insight-like sudden comprehension, whereas the pseudo-solution to control items should induce continued incomprehension. Previous studies (Kizilirmak et al., 2016b, 2019) have shown that in contrast to the control condition, the CRA condition is typically associated with 54–75% of subjectively reported Aha! experiences, characterized by suddenness, confidence, ease, and pleasure. Because of this, we assumed that it is possible to induce insight-like sudden comprehension via CRA items. During memory testing, participants attempted to solve items that had already been presented during encoding (“old items”) and new items, again in random order. When a solution was provided by the participant, the participant was asked to say whether they had experienced an Aha! moment as defined by a written definition they had received during instruction [core aspects being: suddenness, surprise, being convinced of the correctness, positive affect, as suggested by Topolinski and Reber (2010)]. We assessed the following dependent variables:

(1) congruency of plausibility decision: proportion of solvable CRA items correctly identified as plausible and unsolvable control items correctly identified as implausible;
(2) indirect memory performance: difference between solution rate of *old* items (= proportion of correctly solved old items) and *new* items (= proportion of correctly solved new items);
(3) direct memory performance:

(a) difference between hit rate of *old* items (= proportion of correctly recognized *old* items) and false alarm rate (= proportion of *new* items incorrectly identified as *old*);
(b) confidence rating from “sure *old*,” “probably *old*,” over “don’t know” to “probably *new*,” and “sure *new*”;

(4) proportion of Aha! experience: subjective feeling of Aha! assessed as a binary measure on day 2 for old and new correctly and incorrectly solved (CRA) items.

The occurrence of a subjective Aha! experience was only assessed on day 2, because the experiment was designed in a way that participants did not have enough time to solve items on day 1 to enable the induction of an insight by presenting the solution.

### Statistical Data Analysis

Preprocessing of the data as well as visualization was performed using SPSS version 25 (IBM, Armonk, NY, United States). Data were analyzed using R version 3.6.2 and RStudio version 1.2.5033 for linear mixed-effects models in case of the recognition memory data, and JASP version 0.11.1 (JASP Team, 2020) for Bayesian model comparisons. The dependent variables proportion of congruent plausibility responses, solution rates (indirect memory performance), and Aha! rates were all analyzed by means of Bayesian model comparisons (see Section “Bayesian Model Comparisons”). More details on the included terms can be found in the respective *Results* subsections.

The recognition memory ratings (direct memory performance) were analyzed in two ways: (1) using linear mixed-effects models as implemented in R’s lme4 package version 1.1-21 (Bates et al., 2015) to analyze the confidence rating, and (2) using JASP and Bayesian model comparison on the discrimination rate, computed as hits minus false alarms. In contrast to all other analyses, we needed to analyze the confidence ratings on the single-trial level to not loose information about the distribution of the five response categories “sure new,” “probably new,” “don’t know,” “probably old,” “sure old” coded as –2, –1, 0, 1, 2. Models were fitted using the restricted maximum likelihood (ReML) method. Using summary statistics would have meant a large number of empty cells for several participants and combinations of the included factors *Stimulus* (old, new), *Condition* (CRA, control), *Response* (sure new, probably new, don’t know, probably old, sure old). Therefore, we decided for this comprehensive analysis approach so that all data could be taken into account.

### Bayesian Model Comparisons

We used Bayesian model comparison for ANOVA designs, as implemented in JASP Version 0.11.1 (JASP Team, 2020). In this framework, terms for main effects (e.g., *Condition* or *Age*) can be either included into or excluded from the model; and terms for interaction effects (e.g., *Condition* × *Age*) can be either included into or excluded from the model when all main effect terms which they are based on are present in the model (i.e., *Condition* × *Age* × *Site* can only be varied when *Condition*, *Age* and *Site* are contained in the model, cf. Supplementary Table 1). Moreover, all models (incl. the null model) include *Subject* as a random effect in mixed designs with within-subject factors (e.g., *Condition*).

This leads to a specific number of models for given set of factors (e.g., 19 models for 3 factors, including the null model, cf. Supplementary Table 1). The prior probability for each model is specified as 1 divided by the number of all models. Consequently, main effects, two-way interactions and three-way interactions receive different prior probabilities, because they are contained in a different number of models (see e.g., Table 2). Then, posterior probabilities for all models are calculated and from these, the posterior probability of each effect, i.e., of including the respective term (e.g., the main effect of *Condition*), can be derived (see Supplementary Methods for more details).

**TABLE 2:** Results for all considered effects in the Bayesian model comparison for the congruency decision and mean response times of the associated button press.

	Frequency of congruent responses			Response time		
Effect	*P*(E)	*P*(E|y)	BF_*E*_	*P*(E)	*P*(E|y)	BF_*E*_
Condition	*0.737*	*1.000*	*757.169*	0.600	0.332	0.177
Age	*0.737*	*0.956*	*7.671*	0.600	0.482	0.332
Site	0.737	0.472	0.319			
Age*Condition	*0.316*	*0.925*	*26.860*	0.200	0.037	0.083
Age*Site	0.316	0.114	0.280			
Site*Condition	0.316	0.136	0.340			
Age*Site*Condition	0.053	0.009	0.165			

*Terms are sorted by complexity and posterior probability, and highlighted in *italics* when part of the winning model. *P*(E), prior probability; *P*(E| y), posterior probability; BF_*E*_, Bayes factor favoring inclusion of the effect.*

In the results tables, P(E) and P(E|y) denote the prior and posterior probability of including an effect and BF_*E*_ denotes the Bayes factor in favor of including this effect, which quantifies how much more likely it is to observe the measured data under H1 (in this case, all the models including the term), when compared to observing the data under H0 (all models not including the term). In the text, we additionally report the single winning model when inferring on models rather than effects (see Supplementary Results for more details).

## Results

### Age-Related Vocabulary Differences

We compared the MWT-B sum scores of both age groups via Bayesian ANOVA, including *Age* (young, older) and *Site* (Hildesheim, Magdeburg) as factors. The winning model was the one including just *Age* [prior probability of *P*(M) = 0.200, posterior probability of *P*(M| y) = 0.528], indicating that *Site* had no relevant effect. In line with the literature (Park et al., 2002), older adults performed considerably better (mean = 31.4, *SD* = 2.4) compared with younger adults (mean = 25.0, *SD* = 2.2). The full model space, prior and posterior probabilities are reported in Supplementary Table 1 and the single effects can be found in Supplementary Table 2.

### Congruency of Plausibility Decision and Response Times

**FIGURE 2:**
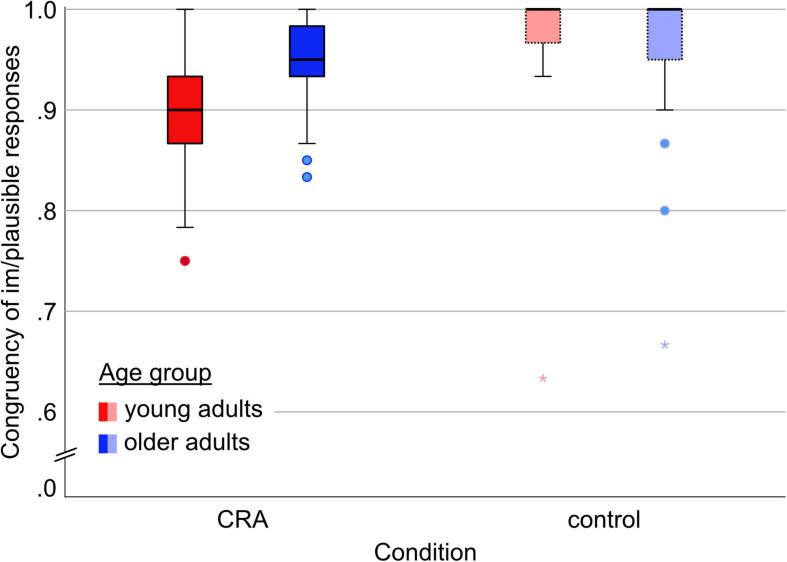
Frequency of congruent responses of the plausibility decision by condition and by age group. Congruent responses: plausible CRA and implausible control.

The plausibility rating was primarily assessed as a control variable, to motivate active processing of the items for incidental encoding. Plausibility (plausible/implausible) was recoded into a variable termed *congruency* (1 for plausible CRA and implausible control, 0 for implausible CRA and plausible control). Older participants provided congruent plausibility answers for 94.8% (*SD* = 4.4%) of all CRA items and 95.6% (7.5%) of all control items. Young participants provided congruent responses for 89.2% (6.1%) of all CRA items and 97.2% (6.8%) of all control items. The whole pattern is depicted in Figure 2.

We included *Age* (younger, older), *Condition* (solvable, unsolvable), and *Site* (Hildesheim, Magdeburg) as well as all possible interaction terms in the Bayesian model comparison with proportion of congruent responses as dependent variable. The winning model comprised main effects of *Age* and *Condition* as well as the interaction term *Age × Condition* [prior probability of *P*(M) = 0.053, posterior probability of *P*(M|y) = 0.485]. *Site* (Magdeburg, Hildesheim) explained a negligible amount of variance [see Table 2. Results for all considered effects in the Bayesian model comparison for the congruency for a comprehensive list of all included effects, Bayes factors (BF), and prior/posterior probability of each effect]. The full model space is reported in Supplementary Table 3.

Responses for control items were more accurate (at ceiling) compared to CRA items. Moreover, while older and young participants performed equally well for control items, the older participants were more accurate when rating CRA items than were the young group (see Figure 2).

We further assessed potential response time (RT) differences, depending on Age (young, older) and Condition (solvable, unsolvable), setting up the model comparison as for the frequency of congruent responses. Incongruent responses, that is, incorrect responses, were excluded from analysis. Unfortunately, all participants from Magdeburg had to be excluded from this analysis, because – with the exception of four participants – there was a problem with logging RTs for the right arrow key (plausible response for even numbered participants and implausible response for odd numbered participants).

The winning model was actually the null model [*P*(M) = 0.200, *P*(M|y) = 0.344; see Table 2. Results for all considered effects in the Bayesian model comparison for the congruency for a list of all considered effects and Supplementary Table 4 for a report of the full model space with prior and posterior model probabilities, and BFs]. In other words, the data were most likely to be observed under a model just assuming a random effect of participant.

Descriptively, older participants responded more slowly (CRA: mean = 4894 ms, *SD* = 1939 ms; control: 5276 ms, 2303 ms) compared to young participants (CRA: 3989 ms, 2063 ms; control: 4385 ms, 3009 ms), and participants responded more slowly to control items than to CRA items.

### Indirect Memory Performance: Solution Rate on Day 2

As expected, no old or new control items were solved on day 2 (with the exception of one young participant who remembered the pseudo-solution of one unsolvable control item). Hence, we could not include the within-subjects factor *Condition*, but focused the analysis of indirect memory performance on solvable CRA items. We analyzed the proportion of correctly solved *old* and *new* CRA items [within-subjects factor *Stimulus* (old, new)] with *Age* (young, older) and *Site* (Hildesheim, Magdeburg) as between-subjects factors. The winning model which best explains the variance in the observed data [*P*(M) = 0.053, *P*(M| y) = 0.506] was the one including Stimulus, Age and the interaction term Stimulus^∗^Age (see Table 3 for all included terms and Supplementary Table 5 for the whole model space). Again, site explained a negligible amount of variance.

**TABLE 3:** Results for all considered effects in the Bayesian model comparison for solution rate and response times.

	Solution rate			Response time		
Term	*P*(E)	*P*(E| y)	BF_*E*_	*P*(E)	*P*(E| y)	BF_*E*_
*Stimulus*	*0.737*	*1.000*	*1.109e*+*14*	*0.737*	*1.000*	*3.125e*+*7*
*Age*	*0.737*	*1.000*	*137047.575*	*0.737*	*0.892*	*2.949*
Site	0.737	0.493	0.348	*0.737*	*0.766*	*1.171*
Stimulus*Age	*0.316*	*0.999*	*2877.221*	*0.316*	*0.558*	*2.733*
Stimulus*Site	0.316	0.249	0.719	0.316	0.246	0.707
Age*Site	0.316	0.209	0.573	0.316	0.226	0.632
Stimulus*Age*Site	0.053	0.067	1.292	0.053	0.012	0.210

*See Table 2 for detailed legends.*

**FIGURE 3:**
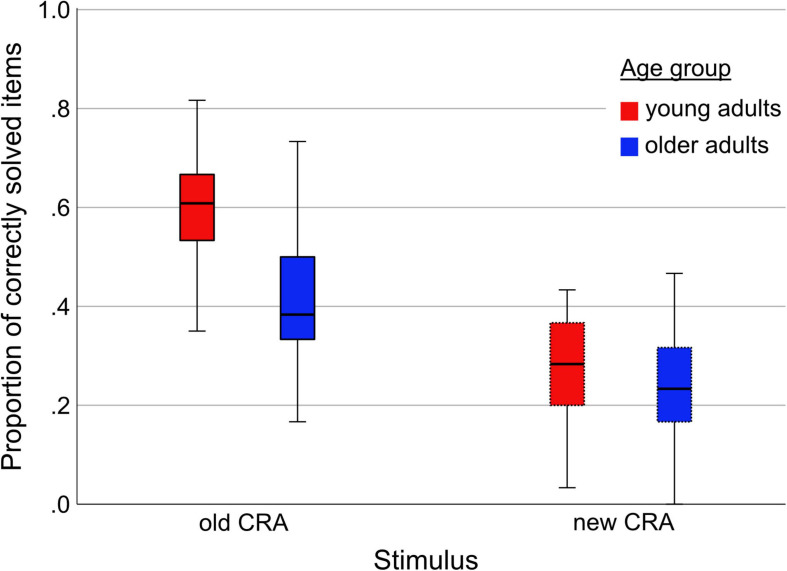
Proportion of correctly solved CRA items by Stimulus (old, new) and Age group (young, older).

Boxplots for all conditions are depicted in Figure 3. Of all solvable CRA items, the older subjects solved a mean of 40.8% (*SD* = 13.3%) *old* items and 23.8% (10.5%) *new* items. The young subjects solved 58.7% (11.7%) *old* items and 27.8% (10.6%) *new* items. As can be seen in Figure 3, young participants benefited considerably more from solving *old* items (mean difference *old-new* = 30.9%, *SD* = 12.2%) compared to the older group (17.0%, 10.2%).

As solution rates could be dependent on reaction times and reaction times are known to slow with increasing age (Bellis, 1933), we also compared reaction times of correctly solved CRA items with regard to *Stimulus* (old, new), *Age* (young, older), and *Site* (Hildesheim, Magdeburg). Please note that a single older participant never solved any *new* item, which is why this subject was not included (see Supplementary Table 6 for full model comparison). The winning model was the one containing main effect terms for *Stimulus, Age*, and *Site*, as well as the interaction term *Stimulus^∗^Age* [*P*(M) = 0.053, *P*(M| y) = 0.202, but see Table 3]. This was the only model comparison in which a model containing *Site* was the best way to explain the observed data.

Participants measured in Magdeburg (MD) had generally longer RTs compared to participants measured in Hildesheim (HI). As displayed taken in Table 4, older and younger participants showed similarly slow RTs for new items, but the younger participants were considerably faster in responding to *old* items, mirroring the solution rates reported above (see Table 4 and Figure 3).

**TABLE 4:** Descriptive statistics for response times of correctly solved CRA items.

						95% Credible Interval	
Stimulus	Age	Site	Mean	*SD*	*N*	Lower	Upper
New	Older	HI	7390	1720	15	6438	8343
		MD	8288	1390	15	7518	9058
	Young	HI	7077	1497	15	6248	7906
		MD	7857	1774	15	6874	8839
Old	Older	HI	6665	1271	15	5961	7369
		MD	7087	987	15	6540	7634
	Young	HI	5616	1122	15	4995	6238
		MD	6007	1055	15	5423	6592

**FIGURE 4:**
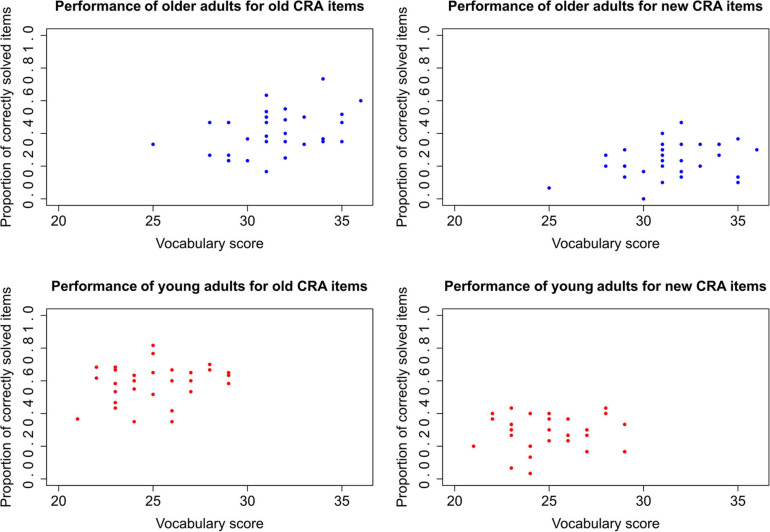
Correlation between score of vocabulary test (MWT-B) and frequency of correctly solved CRA items.

To further assess whether the solution rates of *old* and *new* CRA items were positively correlated with the participants’ vocabulary (this was H1; H0 was that there was no such correlation), we computed Bayesian correlations for each age group and item category. We calculated Pearson’s rho, but, instead of *p*-values and confidence intervals, BFs and credibility intervals were computed to decide which hypothesis is most likely, given the data. The correlations are depicted in Figure 4.

For older participants, there was moderate evidence for a positive correlation for old CRA items (*r* = 0.398, BF_10_ = 4.599). Thus, it is about 4.6 times more likely that there is a positive correlation than that there is none. However, for new CRA items, there is only anecdotal evidence for such a relationship (*r* = 0.308, BF_10_ = 1.642). Thus, there probably is no such correlation for new items.

For younger participants, there was merely anecdotal evidence for the H0 for old CRA items (*r* = 0.226, BF_10_ = 0.795). For new CRA items, there was even moderate evidence for H0, that is, the absence of a positive correlation, which is of no surprise with an *r* = –0.006 (BF_10_ = 0.221).

To summarize, if any correlation can be assumed between vocabulary and CRA solution frequency, then only for older participants and old CRA items.

### Direct Memory Performance: Recognition of Old Items on Day 2

To address the main hypothesis, namely an increased beneficial effect from sudden comprehension on later recognition memory in older adults more directly, we ran a simplified analysis, in which all “don’t know” responses were excluded, and the “probably” and “sure” responses were collapsed. This enabled us to calculate classical hit and false alarm rates to infer how well participants could discriminate between old and new items. We computed differences between hit rates and false alarm rates for each condition and ran a Bayesian model comparison including *Age* (older, young), *Site* (Hildesheim, Magdeburg) and *Condition* (CRA, control) as well as all potential interaction terms (see Table 5). The winning model was the one including main effects of *Condition* and *Age* [*P*(M) = 0.053, *P*(M| y) = 0.337; see Supplementary Table 7 for a comprehensive list of all models].

**TABLE 5:** Results for all included terms in the Bayesian model comparison for recognition memory performance (difference hits – misses).

Term	*P*(E)	*P*(E|y)	BF_*E*_
*Condition*	*0.737*	*1.000*	*7.706e*+*9*
*Age*	*0.737*	*1.000*	*12602.900*
Site	0.737	0.357	0.198
Condition*Age	0.316	0.489	2.076
Condition*Site	0.316	0.087	0.207
Age*Site	0.316	0.102	0.245
Condition*Age*Site	0.053	0.004	0.075

*See Table 2 for detailed legends.*

**FIGURE 5:**
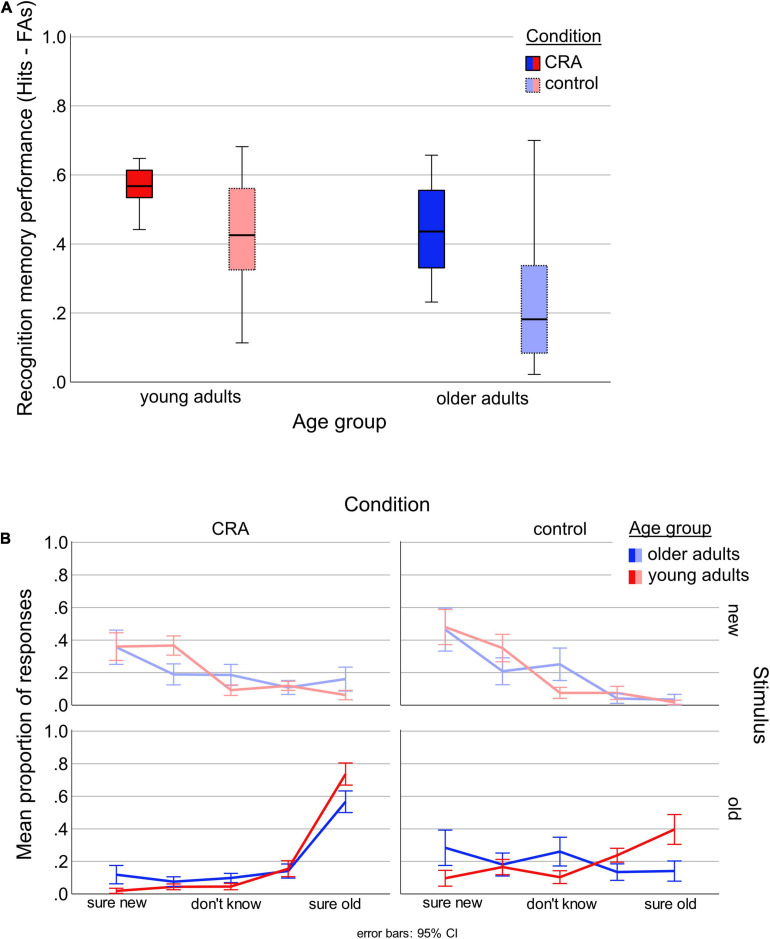
Recognition memory performance. **(A)** Depicts the ability to discriminate between old and new items (proportion of hits minus false alarms) by Condition (solvable CRA, unsolvable control) and Age group (young, older). **(B)** Shows the mean proportion of each response category for the recognition memory rating [sure new (–2), probably new (–1), don’t know (0), probably old (1), sure old (2)] by Condition (CRA, control), Stimulus (old, new), and Age group (young, older).

As can be seen in Figure 5A and Table 6, while the young performed generally better in discriminating between old and new items, the difference between the performances of both age groups was significantly smaller for CRA compared to control items (see for descriptive values). Moreover, old/new discrimination of CRA items in older adults was equal to discrimination of control items in younger adults. From another perspective, while the discrimination rate for the young was 13.6% higher for CRA compared to control, the benefit was 20.7% and thus considerably higher for the older participants (see Table 6 for comprehensive descriptive statistics).

**TABLE 6:** Descriptive statistics for the recognition memory performance (hits-misses).

					95% Credible Interval	
Condition	Age	Mean	*SD*	*N*	Lower	Upper
Control	Older	0.236	0.191	31	0.166	0.306
	Young	0.433	0.141	30	0.380	0.485
CRA	Older	0.443	0.128	31	0.397	0.490
	Young	0.569	0.055	30	0.548	0.589

Recognition memory performance was also analyzed by using linear mixed-effects models to obtain further information about the confidence with which participants made their recognition memory decision. Linear mixed-effects models were used to allow for a trial-wise analysis of the data, which was necessary, because not all participants made use of all available response options in each condition. The latter would have resulted in many participants with empty cells when using classical averaging per participant and per condition. We first computed a null model with only *Subject* (*N* = 61) and *Solutions* (*N* = 182)^4^ as random intercepts for later comparison. This null model (M0) was compared with a model M1 that additionally included the fixed-effects factors *Condition* (CRA, control), *Stimulus* (*old, new*), and *Age* (older, young), a model M2 including also the respective interaction terms, and a model M2a that included *Condition* as random slope to address the individual differences in CRA versus control slopes. All models are listed in Table 7. We ran model comparisons by using R’s anova() function. The winning model was M2a.

**TABLE 7:** Linear mixed-effects models for recognition memory rating as measured on a 5-point scale (–2 “sure new,” –1 “probably new,” 0 “undecided,” 1 “probably old,” and 2 “sure old”).

Model	BIC	Formula
M0	30433	recogn_rating ∼ 1 + (1| subject) + (1| target) + ε
M1	26923	recogn_rating ∼ 1 + condition + age + stimulus + (1| subject) + (1| target) + ε
M2	26476	recogn_rating ∼ 1 + condition + age + stimulus + condition*age + condition*stimulus + age*stimulus + condition*age*stimulus + (1| subject) + (1| target) + ε
M2a	26328	recogn_rating ∼ 1 + condition + age + stimulus + condition*age + condition*stimulus + age*stimulus+condition*age*stimulus + (1 + condition| subject) + (1| target) + ε

*Model formulas are provided in Wilkinson notation. BIC, Bayesian Information Criterion.*

As can be seen in Figure 5B, participants of both age groups were most confident when making their decision on *old* CRA items, while they were less confident when deciding on *old* control items. Moreover, the young made more “probably” responses while older adults made more “don’t know” responses.

Regarding the fixed effects, main effects of *Condition* [*t*(110.34) = 5.09, *p* < 0.001], *Stimulus* [*t*(8027.26) = 30.80, *p* < 0.001], and *Age* [*t*(78.56) = 3.01, *p* = 0.004] were highly significant. The interaction terms *Age*^∗^*Stimulus* [*t*(8082.89) = 14.27, *p* < 0.001] and *Condition*^∗^*Stimulus* [*t*(8103.91) = 9.37, *p* < 0.001] were also highly significant. The interaction between *Condition*^∗^*Age* was not significant [*t*(108.72) = 1.30, *p* = 0.198], whereas the triple interaction reached significance [*t*(8041.06) = 2.01, *p* = 0.044].

### Aha! Experience

To analyze the potential relationship between the proportion of Aha! experiences for correct (true insight) and incorrect solutions (false insight) of *old* and *new* items, we ran another model comparison including between-subjects factor *Age* (young, older), within-subjects factor *Insight* (true, false), within-subjects factor *Stimulus* (*old, new*) and all possible interaction terms (see Table 8). We decided to leave *Site* out of the model, as it had proven largely negligible in the previous model comparisons, and would have made the model space unnecessarily complex. The winning model this time was the simple model only including *Insight* [*P*(M) = 0.058, *P*(M|y) = 0.390; see Supplementary Table 8], suggesting that neither Age nor Stimulus had probable effects, given the data.

**TABLE 8:** Results for all included terms in the Bayesian model comparison for proportion of reported Aha! experiences of incorrectly and correctly solved items.

Term	*P*(E)	*P*(E| y)	BF_*E*_
*Insight*	*0.737*	*1.000*	∞
Stimulus	0.737	0.286	0.143
Age	0.737	0.472	0.319
Insight*Stimulus	0.316	0.075	0.175
Insight*Age	0.316	0.077	0.180
Stimulus*Age	0.316	0.032	0.071
Insight*Stimulus*Age	0.053	0.001	0.020

*See Table 2 for detailed legends.*

**FIGURE 6:**
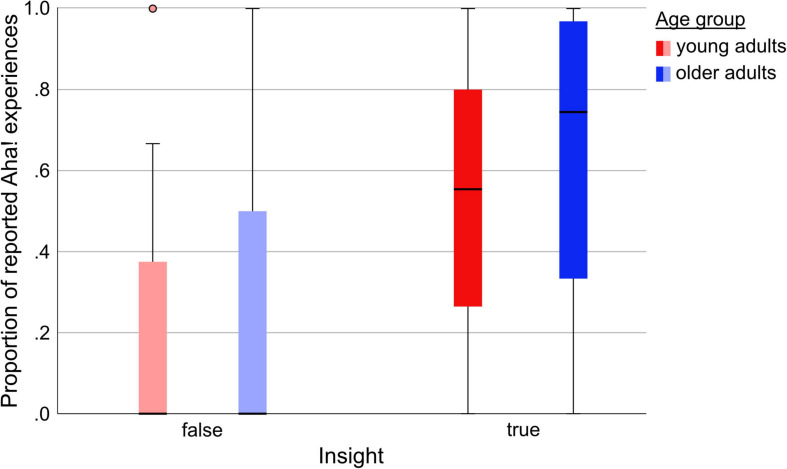
Proportion of reported Aha! experiences for correctly (true insight) and incorrectly (false insight) solved items by Age group (young, older).

As can be seen in Figure 6, true insights were associated with a considerably higher number of Aha! experiences than false insights, and neither the age group (young, older) nor item status (old, new) contributed meaningfully to explaining the observed data (see Table 9 for descriptive statistics).

**TABLE 9:** Proportion of reported Aha! Experiences.

Insight	Stimulus	Age group	Mean	*SD*
True	Old	Older	0.687	0.328
		Young	0.495	0.280
	New	Older	0.599	0.374
		Young	0.583	0.315
False	Old	Older	0.314	0.381
		Young	0.247	0.362
	New	Older	0.256	0.348
		Young	0.139	0.300

## Discussion

This study reports, to our knowledge, the first investigation of age-related differences in problem solving by induced insight-like sudden comprehension and its effects on successful memory formation. The only other study on insight and aging focused on sleep as an incubation period (Debarnot et al., 2017) and did not address learning or memory formation. Our main interest here was, whether learning by induced insight could represent a potential resource in old age. To this end, during memory encoding, we employed an insight-like study condition, which induced sudden comprehension after a previous state of incomprehension (CRA condition), and compared it with a control condition during which the state of incomprehension was maintained. Later memory was tested (1) indirectly by means of solution frequency of old compared to new items (also known as the *re-solution effect;* Dominowski and Buyer, 2000) and (2) directly with regard to the ability to discriminate between old and new items.

### Pronounced Memory Benefit From Sudden Comprehension in Older Adults

In line with our key hypothesis that older adults may show a pronounced memory advantage for CRA items, we found that their recognition memory performance (i.e., their ability to discriminate between *old* and *new* items) strongly benefited from this condition compared to the control condition (see “Direct Memory Performance: Recognition of Old Items on Day 2”). In fact, older adults exhibited a proportionally higher benefit from this insight-like encoding than the young adults. Participants of both age groups further showed a benefit from incidental encoding on the previous day on the indirect measure of memory, with solution rates being generally higher for *old* compared to *new* CRA items (see section “Indirect Memory Performance: Solution Rate on Day 2”). However, this benefit was significantly higher for the young compared to older adults. This effect pattern could either be due to (a) opposing effects of a better ability to solve the riddles in older adults on the one hand (larger vocabulary, Park et al., 2002), but slower processing speed (Salthouse, 1996), or (b) due to better memory for old solutions of the young. The first explanation appears more likely, given the significantly slower RTs of older as compared to young participants for old CRA items, the higher benefit from the CRA condition with respect to discrimination ability in older adults, and the equally high solution rates for new items. However, to evaluate this further, one would need to conduct a follow-up study without timeouts for solving the riddles to avoid potentially confounding effects of solution rates with reaction times or, more generally processing speed.

### Vocabulary Advantage and Higher Accuracy of Plausibility Judgments by Older Adults

Many empirical studies have suggested that older adults have a stronger tendency to generalize information, a phenomenon also associated with increased false-memory effects due to semantic priming (Fandakova et al., 2013), preserved semantic priming even in Alzheimer’s disease (Evrard et al., 2016), reversed self-referential encoding patterns as compared to the young (focusing more on what is common, not the differences) (Gutchess et al., 2010), as well as generalized and reduced task-specific activation patterns in neuroimaging studies (Wang et al., 2010; Hoffman and Morcom, 2018). Yet, at the same time, their vocabulary tends to be more extensive and their semantic knowledge is broader compared to the young, although access is slower and more difficult (Park et al., 2002). Here, we could replicate this finding, using the vocabulary-based MWT-B (Lehrl, 1999), which yielded higher values in our sample of older adults as compared to the young (see section “Age-Related Vocabulary Differences”). Moreover, older adults were better at making plausibility judgments of CRA items (see section “Congruency of Plausibility Decision and Response Times”). The proportion of correctly solved items was not only comparable for *new* items in both age groups, despite the short time limit for attempting to solve the riddles on day 2 (15 s), but also RTs did not differ. This points to the interesting possibility that a broader and more strongly interconnected associative network may exist in the older participants, enabling them to find CRA solutions faster, counteracting the reaction time disadvantage. We found moderate evidence for a positive correlation between vocabulary and solution rates in older participants, but only for old CRA items, while it was only negligible for new items (see section “Indirect Memory Performance: Solution Rate on Day 2”). Thus, this interpretation has to remain tentative.

It is likely that the vocabulary advantage of older adults also facilitated their plausibility judgments. Moreover, older adults apparently depend more strongly on prior knowledge during visual as well as memory-related processing, as recently shown by Wynn et al. (2020). These findings are also in line with previous research from a large cognitive training study in older adults (ACTIVE; Gross et al., 2011), which found that verbal memory was a predictor of everyday problem-solving abilities in healthy older adults (>65 years). Thus, in tasks where prior knowledge and strong, generalized interconnectedness of knowledge represents an advantage, as likely in the present problem-solving task, this automatic reliance on existing knowledge can be beneficial. This supports our hypothesis that verbal knowledge represents a cognitive resource in old age.

### Considerations on the Underlying Neural Basis of Observed Age-Related Differences

We tested the hypothesis that learning by induced insight-like sudden comprehension could represent a potential resource for learning at an advanced age, based on the repeated observation from neuroimaging studies of learning by insight (Ludmer et al., 2011; Kizilirmak et al., 2016b, 2019) that this process is mainly hippocampus-independent. Remarkably, instead of the hippocampus, midline structures like the medial prefrontal cortex and other regions of the so-called Default Mode Network (DMN) appear to play important roles in learning by insight. Especially the normally aged older adults (excluding the so-called successful agers) show subsequent memory effects suggesting that they use an hippocampus-independent network for successful episodic memory encoding that shows considerable overlap with the DMN (Maillet and Rajah, 2014). The question arises, whether this shift could represent the neurocognitive basis for beneficial effects of learning by insight in old age, and will be addressed in a next step.

The advantage of learning by insight for older adults, which is reflected by the higher increase in discrimination ability for the CRA as compared to the control condition, is highly interesting in light of a study that revealed impaired problem-solving ability in older adults, especially those with MTL lesions when compared with healthy young subjects (Sheldon et al., 2011). One of the investigated abilities was that of inferring how a solution state could be reached provided a given problem state (so-called Means-End Problem Solving). This task is very similar to our incidental encoding task, because we also provide a problem (triad) and a solution, while participants have to figure out how the solution word can be used to form compound words to make the plausibility judgment about the solution. Sheldon et al. (2011) reported significantly lower proportions of provided means (i.e., ways to reach the solution) in older adults and in patients with MTL lesions as compared to young healthy adults. This finding challenges our hypothesis that older adults may have an advantage in learning via insight due to it being less hippocampus-dependent. If problem solving depends on the MTL and especially the hippocampus, as not only Sheldon et al. but also others suggest for insight problem solving in particular (Luo and Niki, 2003; Kizilirmak et al., 2016b), how can it be that learning by insight is still enhanced as supported by facilitating old/new discrimination? This question can only be answered by neuroimaging studies that look further into age-related differences in neural networks of memory formation during learning by insight. Studies with young healthy participants at least suggest that while the hippocampus is indeed involved in insight-like sudden comprehension during problem solving, it plays no significant role in successful learning by insight (Ludmer et al., 2011; Kizilirmak et al., 2016b, 2019). Instead, the medial prefrontal cortex, which has been shown to play a key role in prior-knowledge dependent encoding (van Kesteren et al., 2012, 2013), seems relevant for this type of memory encoding.

### Limitations

There are a number of limitations on the type of inferences we can draw from the current study. One is with regard to the operationalization of insight, or, more precisely, induced insight-like sudden comprehension. We did not assess, during encoding, whether participants actually had an “Aha!” experience concurrent with comprehending the presented solution to CRA problems. We refrained from adding a Aha!/no Aha! decision to the important plausibility judgment, because we intend to use the same design not only with functional magnetic resonance imaging, but also with clinical populations for whom the cognitive load of the task should be kept at a minimum (e.g., Alzheimer’s disease). Nevertheless, because of this, we can only make indirect inferences on whether the CRA condition was indeed a true *insight* condition – on the one hand, from the memory test of the current study, where we did assess the Aha experience, and, on the other hand, from other studies, in which we generally used the same encoding task, but also assessed Aha! in addition to plausibility (Kizilirmak et al., 2016b, 2019). From the memory test, we know that on average participants reported 59.2% of all correctly solved (old and new) CRA items to be associated with a concurrent subjective feeling of Aha!. From the named functional neuroimaging studies, we found that, during encoding, participants reported between 54 and 75% of Aha! experiences for presented CRA solutions. Thus, there is a high likelihood that in the majority of trials, participants did indeed comprehended the solutions to CRA with insight-like sudden comprehension or Aha!.

Another important consideration that has to be made is the question whether the here-used operationalization of insight and its beneficial effect on later old/new discrimination especially for the older adults is actually something special. The CRA can be understood as a sudden comprehension condition, whereas the control condition is a continued incomprehension condition. Here, we defined this “initial non-comprehension followed by comprehension” as *insight* (Auble et al., 1979). It has previously been shown in a series of experiments that this condition is more beneficial to later memory than (1) immediate comprehension and (2) continued incomprehension (Auble and Franks, 1978; Auble et al., 1979). The results of these studies further suggest that “the amount of elaborative processing does not seem to be an effective determinant of later recall” (Auble et al., 1979, p. 433) – neither when amount of elaboration was operationalized as “time to elaborate” nor as “difficulty to comprehend”. According to this line of thought, we compared response times for the plausibility response on day 1 (see section “Congruency of Plausibility Decision and Response Times”). Indeed, RTs were longer for the control condition, both in young and older adults. Moreover, in both conditions, participants had the exact same instructions during encoding: Try to understand whether and how the solution word can be used to build compound words with each of the other three. Thus, participants performed a semantic (or at least lexical) elaboration task on all items. We therefore conclude that *changing a state of incomprehension into comprehension* is of key relevance to learning in this context. At least elaboration in the sense of effort toward comprehension was not they key to facilitate learning.

One question that arises is whether our conditions (CRA versus control) could be compared with deep versus shallow levels of processing (Craik and Lockhart, 1972; Eysenck, 1979; Craik, 2002). Before the presentation of the solution, this is most definitely not the case. In both conditions, three words are presented that, on first glance, do not seem to be semantically related. Participants search for the compound solution word lexically and semantically. Levels of processing should be about equal to this point. After presentation of the solution, the link between the triad words becomes evident by means of the presented solution for CRA items only (=insight-like sudden comprehension), but not for control items. Based on these considerations, one could say that there is some difference with regard to the levels-of-processing, because CRA items can more easily be linked lexically (and to some degree semantically) than control items.

Another question is whether the results be explained by prior knowledge related versus prior knowledge unrelated encoding differences (van Kesteren et al., 2014; Greve et al., 2019; Wade and Kidd, 2019). Both conditions work with the exact same words (see section “Stimulus Material”), so there should be no general vocabulary differences. However, in the CRA condition, the words that make up the items can be linked based on prior knowledge, that is, by comprehending the valid compound words and their association via the solution word. Thus, there is a difference with regard to the benefit of prior knowledge on encoding.

To summarize, the here studied insight-like sudden comprehension appears to combine several phenomena (levels-of-processing, prior knowledge based encoding) that are each on their own known to be beneficial to memory encoding, and especially so for older participants (Kan et al., 2020; Ryan et al., 2020). We would like to propose that this combined effect, plus the key aspect of changing a state of incomprehension into sudden comprehension makes the insight memory advantage special in its own way.

### Clinical Implications

Age-related memory impairment, while physiologically observed, must be differentiated from the early, pre-clinical stages of Alzheimer’s disease (AD). The gross anatomical changes in AD, which are observed with brain imaging, are preceded by accumulations of pathological proteins in the brain, most prominently plaques formed by beta-amyloid (Aβ) and neurofibrillary tangles consisting of aggregated tau protein (Schapira et al., 2017). While Aβ deposition correlates poorly with cognitive abnormalities, tau deposition is mirrored by characteristic cognitive deficits (Maass et al., 2019). In early AD, tau begins to accumulate in the MTL and is highly related to MTL atrophy and a deficit in object memory. Subsequently, tau pathology spreads toward frontal and posterior midline regions (Schöll et al., 2016), and this is reflected by an increasing deficit in scene memory. With respect to the task used here, would thus be highly interesting to study learning by insight in older adults at risk for AD, as indexed by subjective cognitive decline (SCD; Jessen et al., 2020) and biomarkers of neurodegeneration. We predict that older adults at risk for AD with tau pathology restricted to the MTL, would show at least some degree of preserved learning from insight, while performance should largely break down with the spread toward midline brain regions that seem to play a key role in this type of learning (Kizilirmak et al., 2019).

## Conclusion

The results of the current study reveal that healthy older adults can outperform young adults with regard to their ability in judging the plausibility of provided solutions to verbal riddles. Moreover, despite only a brief time for problem-solving, older adults were similarly good as the young in solving novel riddles. Lastly, older adults benefited considerably from learning by insight-like sudden comprehension with regard to later ability to discriminate between *old* and *new* items. This suggests that learning by insight, which relies strongly on prior knowledge, constitutes a promising approach to improve learning and memory performance in old age. The neural underpinnings of this process are yet to be elucidated, and neuroimaging studies are warranted to assess the underlying neural mechanisms proposed in this study.

## Data Availability Statement

The datasets presented in this study can be found in online repositories. The names of the repository/repositories and accession number(s) can be found below: Open Science Framework, https://osf.io/m4w9j.

## Ethics Statement

The studies involving human participants were reviewed and approved by the Ethics Committees of the Department of Education at the University of Hildesheim and of the Otto von Guericke University of Magdeburg, Faculty of Medicine. The participants provided their written informed consent to participate in this study.

## Author Contributions

JK: conceptualization with helpful input from BS, programming of experiment, statistical analysis with helpful input from JS, visualization with helpful suggestions from AR and BS, and writing – original draft. JK and LF: data acquisition. BS: funding acquisition. JK, LF, AR, BS, and JS: writing – revisions. JK and JS: supplement. All authors contributed to the article and approved the submitted version.

## Conflict of Interest

The authors declare that the research was conducted in the absence of any commercial or financial relationships that could be construed as a potential conflict of interest.

## Footnotes

1 At this point we would like to point out that the relative volume loss of the hippocampus is about twice to thrice compared to that of prefrontal regions like the inferior frontal gyrus (Fjell et al., 2009).

2 Please note that, whenever we use italics for “old,” we refer to *old* items to avoid mix-ups with older adults.

3 The sample size was determined by prior experience about effects of the insight memory advantage (Kizilirmak et al., 2016b, 2019). We did not conduct an *a priori* power analysis for this study. Bayesian analyses work fundamentally different from classical null hypothesis significance testing (e.g., Wagenmakers et al., 2016; Faulkenberry et al., 2020; van Doorn et al., 2020). Because unlike Frequentist statistics, Bayesian inference does not control the type I error rate, but quantifies the evidence for competing hypotheses given observed data, there is no possibility to calculate statistical power given significance level and sample size. Instead, Bayesian inference works by updating posterior beliefs about the hypotheses based on the data, and it is even possible to quantify how the posterior probabilities for the hypotheses change with accumulating data. As van Doorn et al. (2020, p. 4) put it: “As may be expected from a framework for the continual updating of knowledge, Bayesian inference allows researchers to monitor evidence as the data come in, and stop whenever they like, for any reason whatsoever. Thus, strictly speaking there is no Bayesian need to pre-specify sample size at all.”

4 Checking the participants’ solutions had led us to accepting two additional alternative solutions for two items.
