# Supplementary material for: Learning by Insight-Like Sudden Comprehension as a Potential Strategy to Improve Memory Encoding in Older Adults
Source: Front Aging Neurosci. 2021 Jun 14;13:661346. doi: 10.3389/fnagi.2021.661346 (PMC8236646; doi:10.3389/fnagi.2021.661346)
Supplement: Supplementary file 1 [file Presentation_1.pdf]

Supplementary Information

for

Learning by insight-like sudden comprehension as a potential resource for learning in advanced age

Jasmin M. Kizilirmak*[1,2], Larissa Fischer[3], Justus Krause[2], Joram Soch[1,4], Anni Richter[3] & Björn H. Schott[1,3,5]

1) German Center for Neurodegenerative Diseases, Von-Siebold-Star. 3a, 37075 Göttingen, Germany

2) Institute for Psychology, University of Hildesheim, Germany

3) Leibniz Institute for Neurobiology, Magdeburg, Germany

4) Bernstein Center for Computational Neuroscience, Berlin, Germany

5) Department of Psychiatry and Psychotherapy, University Medical Center Göttingen, Göttingen, Germany

***Correspondence** should be addressed to JMK, jasmin.kizilirmak@dzne.de

1 Supplementary Methods

Bayesian model selection for ANOVA designs deals with the problem of deciding for an optimal model *M*, given data *y* from a factorial design, specified as a combination of terms for main effects, terms for interactions and terms for higher-level interactions.

For example, if there is a within-subject factor *Condition* and a between-subject factor *Age*, then the possible terms include main effects for *Condition* and *Age*, an interaction effect *Condition* x *Age* and *Subject* as a random effect in mixed models.

When performing Bayesian model comparison for ANOVA designs in JASP (JASP Team, 2020), (i) all models (incl. the null model) include the random effect; (ii) terms for main effects can be either included or excluded from the model; and (iii) terms for interaction effects can be either included or excluded from the model when all main effect terms which they are based on are present in the model (see e.g. Table S1). This leads to a specific number of models for given set of factors (e.g., 19 models for 3 factors, including the null model, cf. Table S1).

Each model is assumed to be equally likely *a priori*, such that the prior probability of each model *M* is specified as 1 divided by the number of models N_M:

$$p(M) = \frac{1}{N_M}$$

Bayesian model assessment is based on the marginal likelihood, i.e., the probability of the observed data *y* given only the model, where model parameters θ have been eliminated from the likelihood function by integrating over the entire parameter space Θ using a prior distribution:

$$p(y|M) = \int_{\Theta} p(y|\theta, M)\, p(\theta|M)\ \mathrm{d}\theta$$

By combining the marginal likelihood with the prior probability, the posterior probability of each model can be calculated using Bayes' theorem:

$$p(M|y) = \frac{p(y|M)\, p(M)}{p(y)} = \frac{p(y|M)\, p(M)}{\sum_{M'} p(y|M')\, p(M')}$$

When selecting from all models, the optimal or "winning" model is considered as the one which maximizes posterior model probability. When comparing just two models, Bayes factors (BF) are calculated as the ratio of the marginal likelihoods of these models:

$$\mathrm{BF}_{ij} = \frac{p(y|M_i)}{p(y|M_j)}$$

In the case of constant prior probabilities across models (see above), a BF is equivalent to the posterior odds ratio, i.e., the ratio of posterior model probabilities:

$$\mathrm{BF}_{ij} = \frac{p(M_i|y)}{p(M_j|y)}, \text{ if } p(M_i) = p(M_j)$$

Here, two Bayes factors are especially important, namely (i) the Bayes factor in favor of each model against the null model, termed BF_{10}, and (ii) the Bayes factor in favor of each model against all the other models, termed BF_M:

$$\mathrm{BF}_{10} = \frac{p(M|y)}{p(M_0|y)}$$

$$\mathrm{BF}_M = \frac{p(M|y)/p(M)}{[1-p(M|y)]/[1-p(M)]}$$

In the case of constant prior probabilities across models, the latter BF can also be expressed as the ratio of a posterior model probability to the average posterior probability of all other models:

$$\mathrm{BF}_M = \frac{p(M|y)}{\frac{1}{N_M - 1}\sum_{M' \neq M} p(M'|y)}, \text{ if } p(M_i) = \frac{1}{N_M} \text{ for all } i = 1, \ldots, N_M$$

Because a model space can become quite complex very fast, even when restricting terms for interaction effects (see above), it is often more desirable to quantify the evidence in favor of *effects E* (e.g., the main effect of one factor) instead of looking at single *models M* (i.e., a particular combination of main effects and interactions). Together, the models from a given model space are typically considered mutually exclusive and collectively exhaustive, such that prior and posterior *effect* probabilities are simply sums of prior and posterior *model* probabilities for all models including the corresponding term:

$$p(E) = \sum_{E \in M'} p(M')$$

$$p(E|y) = \sum_{E \in M'} p(M'|y)$$

Consequently, main effects, two-way interactions and three-way interactions receive different prior probabilities, because they are contained in a different number of models (see e.g. Table 2). Based on the posterior probability for effects, one can finally calculate "inclusion BFs" by contrasting the

posterior probability of including vs. not including a particular term, weighted by the corresponding prior probabilities:

$$\mathrm{BF}_E = \frac{p(E|y)/p(E)}{p(\bar{E}|y)/p(\bar{E})} = \frac{p(E|y)/p(E)}{[1 - p(E|y)]/[1 - p(E)]}$$

To facilitate optimal interpretation of results, we have decided (i) to report $p(E)$, $p(E|y)$ and BF_E for all effects in each analysis in the main manuscript, and (ii) to report $p(M)$, $p(M|y)$, BF_M and BF_{10} for all models in each analysis in this supplementary material. Supplementary Tables additionally report "error %" which is the percentage of possible error when numerically approximating the marginal likelihood – a value that should not exceed 20% (van den Bergh et al., 2020).

2 Supplementary Results

2.1 Age-related Vocabulary Differences

Table S1. Bayesian model comparison for MWT-B sum scores (vocabulary)

Model	P(M)	P(M|y)	BF_M	BF_{10}	error %
Null model	*0.200*	*3.172e -13*	*1.269e -12*	*1.000*	
**Age**	**0.200**	**0.528**	**4.474**	**1.664e +12**	**7.148e -17**
Age + Site + Age*Site	0.200	0.279	1.548	8.794e +11	1.426
Age + Site	0.200	0.193	0.957	6.086e +11	2.535
Site	0.200	9.960e -14	3.984e -13	0.314	0.002

Note. All models include subject

Table S2. Results for all considered effects of the model comparison for MWT-B sum scores

Effect	P(E)	P(E|y)	BF_E
Age	0.600	1.000	1.602e +12
Site	0.600	0.472	0.596
Age*Site	0.200	0.279	1.548

2.2 Congruency of plausibility decision (day 1)

Table S3. Bayesian model comparison for congruence of plausibility decision

Model	P(M)	P(M|y)	BF_{M}	BF_{10}	error %
Null model (incl. subject)	*0.053*	*2.033e -4*	*0.004*	*1.00*	
**Condition + Age + Condition*Age**	**0.053**	**0.485**	**16.956**	**2385.91**	**2.00**
Condition + Age + Site + Condition*Age	0.053	0.239	5.645	1174.31	1.84
Condition + Age + Site + Condition*Age + Condition*Site	0.053	0.090	1.790	444.94	4.45
Condition + Age + Site + Condition*Age + Age*Site	0.053	0.074	1.437	363.64	3.25
Condition + Age + Site + Condition*Age + Condition*Site + Age*Site	0.053	0.028	0.520	138.14	4.80
Condition	0.053	0.027	0.492	131.00	1.70
Condition + Age	0.053	0.016	0.296	79.66	1.91
Condition + Site	0.053	0.013	0.239	64.55	5.53
Condition + Age + Site + Condition*Age + Condition*Site + Age*Site+ Condition*Age*Site	0.053	0.009	0.165	44.65	4.10
Condition + Age + Site	0.053	0.008	0.140	37.92	1.97
Condition + Site + Condition*Site	0.053	0.004	0.080	21.83	2.60
Condition + Age + Site + Condition*Site	0.053	0.003	0.050	13.75	2.73
Condition + Age + Site + Age*Site	0.053	0.002	0.044	11.88	5.13
Condition + Age + Site + Condition*Site + Age*Site	0.053	8.611e -4	0.016	4.24	4.49
Age	0.053	1.160e -4	0.002	0.57	3.65
Site	0.053	8.701e -5	0.002	0.43	0.79
Age + Site	0.053	4.922e -5	8.860e -4	0.24	2.00
Age + Site + Age*Site	0.053	1.590e -5	2.861e -4	0.08	1.84

Note. All models include subject

Table S4. Bayesian model comparison for mean RT of congruent responses for the plausibility decision (only data from Hildesheim due to RT logging error of right arrow button presses)

Models	P(M)	P(M|y)	BF_M	BF_{10}	error %
Null model (incl. subject)	***0.200***	***0.344***	***2.100***	***1.000***	
Age	0.200	0.253	1.355	0.735	1.944
Condition	0.200	0.204	1.027	0.593	2.306
Condition + Age	0.200	0.149	0.703	0.434	6.861
Condition + Age + Condition*Age	0.200	0.049	0.206	0.143	4.134

Note. All models include subject

2.3 Indirect memory performance (day 2)

Table S5. Bayesian model comparison for solution rate of solvable CRA items on day 2

Models	P(M)	P(M|y)	BF_M	BF_{10}	error %
Null model (incl. subject)	*0.053*	*4.641e -26*	*8.354e -25*	*1.00*	
**Stimulus + Age + Stimulus*Age**	**0.053**	**0.506**	**18.457**	**1.091e+25**	**1.95**
Stimulus + Age + Site + Stimulus*Age	0.053	0.163	3.501	3.509e+24	1.90
Stimulus + Age + Site + Stimulus*Age + Stimulus*Site	0.053	0.121	2.481	2.61e+24	1.64
Stimulus + Age + Site + Stimulus*Age + Age*Site	0.053	0.081	1.587	1.746e+24	7.63
Stimulus + Age + Site + Stimulus*Age + Stimulus*Site + Age*Site + Stimulus*Age*Site	0.053	0.067	1.292	1.443e+24	6.74
Stimulus + Age + Site + Stimulus*Age + Stimulus*Site + Age*Site	0.053	0.061	1.169	1.315e+24	4.44
Stimulus + Age	0.053	4.393e-4	0.008	9.465e+21	1.77
Stimulus + Age + Site	0.053	1.261e-4	0.002	2.716e+21	3.27
Stimulus + Age + Site + Stimulus*Site	0.053	1.047e-4	0.002	2.256e+21	21.18
Stimulus + Age + Site + Age*Site	0.053	4.822e-5	8.680e-4	1.039e+21	3.80
Stimulus + Age + Site + Stimulus*Site + Age*Site	0.053	3.160e-5	5.689e-4	6.81e+20	3.73
Stimulus	0.053	1.721e-6	3.097e-5	3.707e+19	1.29
Stimulus + Site	0.053	5.549e-7	9.989e-6	1.196e+19	2.88
Stimulus + Site + Stimulus*Site	0.053	3.304e-7	5.948e-6	7.12e+18	1.73
Age	0.053	8.715e-25	1.569e-23	18.78	1.17
Age + Site	0.053	1.971e-25	3.548e-24	4.25	5.61
Age + Site + Age*Site	0.053	5.857e-26	1.054e-24	1.26	2.18
Site	0.053	1.079e-26	1.943e-25	0.23	5.42

Note. All models include subject

Table S6. Model comparison for response times of correctly solved items (day 2)

**Models**	**P(M)**	**P(M|y)**	**BF_M**	**BF_{10}**	**error %**
Null model (incl. subject)	*0.053*	*1.529e-9*	*2.753e-8*	*1.000*	
**Stimulus + Age + Site + Stimulus*Age**	**0.053**	**0.202**	**4.566**	**1.323e+8**	**11.722**
Stimulus + Age + Site	0.053	0.120	2.449	7.831e+7	3.457
Stimulus + Age + Stimulus*Age	0.053	0.115	2.335	7.510e+7	3.330
Stimulus + Age + Site + Stimulus*Age + Age*Site	0.053	0.100	2.002	6.545e+7	25.395
Stimulus + Age + Site + Stimulus*Age + Stimulus*Site	0.053	0.086	1.697	5.634e+7	4.265
Stimulus + Age	0.053	0.084	1.657	5.514e+7	5.773
Stimulus + Age + Site + Stimulus*Site	0.053	0.059	1.124	3.843e+7	2.421
Stimulus + Site	0.053	0.050	0.942	3.253e+7	3.956
Stimulus + Age + Site + Age*Site	0.053	0.048	0.915	3.165e+7	2.043
Stimulus + Age + Site + Stimulus*Age + Stimulus*Site + Age*Site	0.053	0.043	0.806	2.801e+7	15.824
Stimulus	0.053	0.035	0.644	2.258e+7	1.047
Stimulus + Site + Stimulus*Site	0.053	0.024	0.438	1.554e+7	3.551
Stimulus + Age + Site + Stimulus*Site + Age*Site	0.053	0.023	0.423	1.500e+7	4.058
Stimulus + Age + Site + Stimulus*Age + Stimulus*Site + Age*Site + Stimulus*Age*Site	0.053	0.012	0.210	7.545e+6	3.415
Age + Site	0.053	3.583e-9	6.449e-8	2.343	1.356
Age	0.053	3.208e-9	5.774e-8	2.098	7.016
Site	0.053	1.791e-9	3.224e-8	1.171	1.840
Age + Site + Age*Site	0.053	1.318e-9	2.373e-8	0.862	5.548

Note. All models include subject

2.4 Direct memory performance (day 2)

Table S7. Model comparison for recognition memory performance (hits-misses) on day 2

Model	P(M)	P(M|y)	BF_M	BF_{10}	error %
Null model (incl. subject)	*0.053*	*1.008e -14*	*1.814e -13*	*1.000*	
**Condition + Age**	**0.053**	**0.337**	**9.149**	**3.344e+13**	**8.282**
Condition + Age + Condition*Age	0.053	0.306	7.933	3.036e+13	2.036
Condition + Age + Site + Condition*Age	0.053	0.098	1.963	9.757e+12	2.454
Condition + Age + Site	0.053	0.097	1.938	9.647e+12	2.628
Condition + Age + Site + Condition*Age + Age*Site	0.053	0.038	0.716	3.798e+12	4.625
Condition + Age + Site + Age*Site	0.053	0.036	0.674	3.580e+12	1.671
Condition + Age + Site + Condition*Age + Condition*Site	0.053	0.031	0.573	3.059e+12	5.659
Condition + Age + Site + Condition*Site	0.053	0.029	0.541	2.896e+12	3.366
Condition + Age + Site + Condition*Age + Condition*Site + Age*Site	0.053	0.012	0.217	1.181e+12	3.794
Condition + Age + Site + Condition*Site + Age*Site	0.053	0.011	0.204	1.111e+12	3.345
Condition + Age + Site + Condition*Age + Condition*Site + Age*Site + Condition*Age*Site	0.053	0.004	0.075	4.101e+11	4.526
Condition	0.053	1.928e-5	3.470e-4	1.913e+9	1.246
Condition + Site	0.053	6.949e-6	1.251e-4	6.897e+8	2.108
Condition + Site + Condition*Site	0.053	2.110e-6	3.797e-5	2.094e+8	2.898
Age	0.053	3.424e-11	6.164e-10	3398.472	0.819
Age + Site	0.053	9.170e-12	1.651e-10	910.092	7.213
Age + Site + Age*Site	0.053	2.918e-12	5.253e-11	289.602	3.318
Site	0.053	2.727e-15	4.908e-14	0.271	0.691

Model	P(M)	P(M|y)	BF_M	BF_{10}	error %

Note. All models include subject

2.5 Aha! experience (day 2)

Table S8. Model comparison for relative frequency of Aha! experiences for correctly (true insight) and incorrectly (false insight) CRA items on day 2

Models	P(M)	P(M|y)	BF_M	BF_{10}	error %
Null model (incl. subject)	*0.053*	*5.461e -17*	*9.830e -16*	*1.000*	
**Insight**	**0.053**	**0.390**	**11.506**	**7.141e+15**	1.04
Insight + Age	0.053	0.272	6.737	4.987e+15	2.65
Insight + Stimulus	0.053	0.103	2.067	1.886e+15	2.25
Insight + Stimulus + Age	0.053	0.072	1.392	1.314e+15	3.68
Insight + Age + Insight*Age	0.053	0.052	0.983	9.487e+14	2.76
Insight + Stimulus + Insight*Stimulus	0.053	0.035	0.652	6.398e+14	2.11
Insight + Stimulus + Age + Insight*Stimulus	0.053	0.026	0.473	4.686e+14	7.47
Insight + Stimulus + Age + Stimulus*Age	0.053	0.019	0.345	3.443e+14	3.45
Insight + Stimulus + Age + Insight*Age	0.053	0.014	0.249	2.502e+14	4.88
Insight + Stimulus + Age + Insight*Stimulus + Stimulus*Age	0.053	0.007	0.122	1.230e+14	3.73
Insight + Stimulus + Age + Insight*Stimulus + Insight*Age	0.053	0.005	0.095	9.608e+13	5.95
Insight + Stimulus + Age + Insight*Age + Stimulus*Age	0.053	0.004	0.067	6.841e+13	3.72
Insight + Stimulus + Age + Insight*Stimulus + Insight*Age + Stimulus*Age	0.053	0.001	0.024	2.391e+13	3.91
Insight + Stimulus + Age + Insight*Stimulus + Insight*Age + Stimulus*Age + Insight*Stimulus*Age	0.053	0.001	0.020	2.032e+13	11.71
Age	0.053	3.057e-17	5.503e-16	0.560	0.85
Stimulus	0.053	1.268e-17	2.282e-16	0.232	5.14
Stimulus + Age	0.053	6.884e-18	1.239e-16	0.126	4.40
Stimulus + Age + Stimulus*Age	0.053	1.739e-18	3.130e-17	0.032	4.54

**Models**	**P(M)**	**P(M|y)**	**BF_M**	**BF_{10}**	**error %**

Note. All models include subject

3 References

JASP Team (2020). JASP (Version 0.11.1.0).

van den Bergh, D., van Doorn, J., Marsman, M., Draws, T., van Kesteren, E.-J., Derks, K., et al. (2020). A Tutorial on Conducting and Interpreting a Bayesian ANOVA in JASP. *L'Année Psychol.* 120, 73. doi:10.3917/anpsy1.201.0073.
